# Now is the time for conservationists to stand up for social justice

**DOI:** 10.1371/journal.pbio.3002657

**Published:** 2024-06-10

**Authors:** E. J. Milner-Gulland

**Affiliations:** Department of Biology, University of Oxford, Oxford, United Kingdom; Arizona State University, UNITED STATES

## Abstract

Conservationists are still grappling with what social justice means in practice. This Perspective argues that a major shift in mindset is required in order to achieve socially just, rights-based conservation, and that shift needs to happen now.

## Teaser

Existing power imbalances and injustices could be exacerbated by large flows of international funding for nature recovery. Conservationists are still grappling with what social justice means in practice; a major shift in mindset is required.

Under the Convention on Biological Diversity’s (CBD) Kunming–Montreal Global Biodiversity Framework (GBF), countries agreed to halt and reverse the loss of biodiversity by 2030, towards a world living in harmony with nature by 2050. This ambitious vision requires nature restoration on a grand scale, guided by a slew of data at the local, national, and global levels. The negative impacts on people and nature of nations’ home and overseas biodiversity footprints must be quantified, as must the gains from actions undertaken to address these impacts and contribute to nature recovery. The terms “biodiversity” and “nature” are often used interchangeably. Here, I use “nature” when I mean the natural environment within which we all live and which we value, and I use “biodiversity” when it is part of a widely used term (e.g., biodiversity credit) or to mean an element of nature which is being quantified.

Nature recovery will require large-scale flows of finance from business and government (mostly in the wealthy Global North) to areas where nature destruction is occurring (mostly in poor rural areas of the tropics) [1]. Markets are being generated by those seeking to offset their carbon and biodiversity impacts or invest in biodiversity credits; biodiversity finance also flows through bilateral and multilateral structures including overseas aid, loss and damage funds, Global Environment Facility funding, and traditional conservation avenues. International financial flows related to carbon are much larger and more established than those for nature. Much carbon finance flows to interventions to retain or sequester carbon via avoidance of further loss of natural habitat or restoration of degraded land. Major concerns have been raised about these flows, not just about the effectiveness of nature-based investments as a mechanism for carbon offsetting, but also about human rights violations related to these investments. However, markets to trade gains and losses in greenhouse gases are conceptually simpler than markets for nature, because nature is not fungible; it is dynamic and multi-scale in time and space.

We cannot pick and choose where and what type of biodiversity to protect and restore: it needs to be measured and recovered locally to where the impact is. Therefore, no single composite metric (like tCO_2_e for greenhouse gases) can capture the complexity integral to functioning natural ecosystems, allowing losses in one context to be offset by gains in another. However, we also need to aggregate information on biodiversity trends across scales to inform national and corporate biodiversity strategies and to measure overall progress towards the GBF’s goal of halting and reversing the loss of biodiversity by 2030. The GBF has a plethora of associated indicators, although some components of biodiversity and drivers of loss are still not well covered (e.g., sustainable use of wildlife). These indicators also do not align well with the indicators being developed for businesses to quantify their biodiversity impacts and guide actions contributing to global Nature Positive goals [2].

Designing and implementing global nature recovery strategies presents challenges beyond designing suitable indicators. Firstly, we face a wicked problem. The goals of recovering nature, stabilizing the climate, and promoting human development interact in complex ways; positive and negative feedbacks abound. Worsening of one dimension exacerbates the others, making it harder to reach the win-win-win nirvana. Secondly, protected areas are not enough. We cannot just focus on managing important areas for biodiversity if we are to halt and reverse the loss of biodiversity; global consumption has to drastically reduce if nature is to recover [3]. Responsibility for impact is unequally distributed, both now and in the past, so rich countries must take the main burden of impact reduction [4]. Thirdly, nature is intimately intertwined with human lives in ways that vary in time and space, requiring locally situated conservation [1]. Social impact assessments cannot just focus on material wellbeing or livelihoods; they must include bottom-up conceptions of what matters to people and how biodiversity-focused interventions affect them [5]. And finally, the global evidence base for planning action to recover nature is patchy and biased, regarding both biodiversity trends and their drivers, as well as knowledge about how best to intervene. Conservation’s record on robust evaluation of the impact of interventions is woeful [6]. This risks inappropriate and ineffective interventions that are geographically and taxonomically biased.

Global conservation discourse often invokes social justice, but too often this means benefit-sharing from protected areas or livelihoods support, with success metrics related to material wellbeing. Distributive equity is regularly discussed (e.g., whether the benefits are fairly divided), but what “fair” means is generally based on the subjective interpretation of the person doing the dividing, rather than on internationally agreed norms or standards. Recognition and procedural equity are much less prominent in discourse and in practice, and human rights even less so.

Good practice does exist of course (Box 1), but involvement of local residents in conservation is currently still predominately post hoc (occurring after the site/approach has been chosen) and reactive (based on social safeguards and grievance mechanisms in case of complaints). External involvement in an area is usually based on the presence of globally valued biodiversity (e.g., relatively intact forest, threatened species, areas with restoration potential). Free, prior, and informed consent is not always sought from local communities for proposed conservation actions, and if it is, this is generally only at the beginning of the process, rather than as part of an ongoing dialogue.

**Box 1. Examples of good practice.**

There are very few examples of flipping the top-down model of Western biodiversity science so that scientists are responding to requests for support. However, in New Ireland (Papua New Guinea), a long-term collaboration of an Indigenous nongovernmental organization, Indigenous scientists, and Western researchers has been established to support marine conservation and fisheries management [7]. The Convention on Biological Diversity’s Local Biodiversity Outlooks has many examples of on-the-ground biodiversity conservation initiatives initiated and led by Indigenous Peoples and Local Communities.

The Transformative Pathways project, led by the Forest Peoples Programme, is supporting Indigenous Peoples to lead, and scale up, conservation and sustainable use of biodiversity. One aim is to enable the contributions of Indigenous Peoples to national and global biodiversity priorities to be better appreciated. This includes supporting Indigenous communities who would like to think through and strengthen their biodiversity monitoring strategies, including integrating traditional and “scientific” monitoring of biodiversity trends.

Several large international NGOs support Indigenous Peoples and Local Communities in gaining formal land rights and defending their lands against external encroachment (often by powerful actors). One example is the Wildlife Conservation Society’s participatory land use planning and support for land titling for communities living in Protected Areas in Cambodia [8]. Another is WWF-Brazil’s support of Indigenous groups fighting to protect the Amazon from encroachment.

A limited, but growing, body of literature provides robust evaluations of the social and biodiversity impacts of biodiversity interventions, including biodiversity offsets. One of the most comprehensive studies is an evaluation of offsets associated with the Ambatovy mine in Madagascar. This includes understanding the heterogeneous social impacts of the mine [9], while also demonstrating that overall, the mine’s offset is on track to deliver no net loss of forest cover [10]. Groupings such as the Society for Conservation Biology’s Impact Evaluation Working Group support learning and sharing of best practice in this space.

If conservationists want to contribute to a more socially just and, ultimately, more sustainable world, the power dynamic needs to flip. We need a bottom-up approach, where large flows of money for biodiversity are mobilized on the basis of locally expressed needs and desires, rather than the largest bang-for-buck for external investors. However, conservationists’ priority to protect globally precious and irreplaceable biodiversity does not fully align with priorities for human development, or even for biodiversity conservation at local or national levels. Currently, the honest and equitable conversations about how priorities and values align or conflict are not taking place. These conversations require conservationists to get off the moral high ground and recognize the trade-offs and synergies implicit in achieving nature, societal, and climate goals.

Based on this analysis, some prerequisites for more socially just conservation could include:

- Ensuring that human rights are a legal obligation and form the fundamental red line underpinning any conservation intervention [11].
- Including recognitional and procedural equity into planning and evaluation of conservation interventions, not just distributive equity.
- Not speaking for people; supporting and empowering them to speak for themselves.
- Exploring ways to be available to support and facilitate residents of biodiverse, vulnerable places to conserve their own nature, rather than following externally generated conservation priorities and then getting them on-side.
- Being prepared to listen to what people say and considering what should happen if their priorities and plans do not align with external agendas.

Deep-rooted mistrust between conservationists and those affected by conservation actions, based on negative past or current experiences, takes time to heal, so conservation will be slower and more difficult if it is done in a socially just way. The conservation sector itself is still Global North dominated; meaningful action to support Global South colleagues to lead at the global, national, and local levels is also required if the sector is to transform.

Conservationists and biodiversity scientists have the power, as a sector, not to participate in the development and use of inadequate, simplistic, and externally derived indicators for biodiversity and social outcomes, which fail to recognize the rights, perspectives, and plural values for biodiversity held by local residents [12]. Instead, we must collaborate to develop biodiversity monitoring frameworks that embed social justice and enable robust design and evaluation of interventions in order to learn. This will require difficult choices to be made, and outcomes will be slower to achieve, but it would be a key contribution towards the CBD’s vision of humanity living in harmony with nature.

Social justice and planetary justice can (and must) align, but this requires those who currently have power and privilege to give some of it up. As international biodiversity markets develop, the pressure to cut corners will only grow. Rights-based, socially just conservation is a rocky path, and particularly tempting to bypass when it seems that large-scale funding for nature is finally being unlocked. But if we do not make a stand now, when the structures, metrics, and approaches for channeling international flows of funding for nature recovery are being developed, inequity will be locked in and ultimately both people and nature will lose.
